# Keratin Retraction and Desmoglein3 Internalization Independently Contribute to Autoantibody-Induced Cell Dissociation in Pemphigus Vulgaris

**DOI:** 10.3389/fimmu.2018.00858

**Published:** 2018-04-25

**Authors:** Elisabeth Schlögl, Mariya Y. Radeva, Franziska Vielmuth, Camilla Schinner, Jens Waschke, Volker Spindler

**Affiliations:** ^1^Chair of Vegetative Anatomy, Faculty of Medicine, Institute of Anatomy, Ludwig Maximilian University of Munich, Munich, Germany; ^2^Department of Biomedicine, University of Basel, Basel, Switzerland

**Keywords:** pemphigus, keratin filaments, desmosome, cell adhesion, keratinocytes

## Abstract

*Pemphigus vulgaris* (PV) is a potentially lethal autoimmune disease characterized by blister formation of the skin and mucous membranes and is caused by autoantibodies against desmoglein (Dsg) 1 and Dsg3. Dsg1 and Dsg3 are linked to keratin filaments in desmosomes, adhering junctions abundant in tissues exposed to high levels of mechanical stress. The binding of the autoantibodies leads to internalization of Dsg3 and a collapse of the keratin cytoskeleton—yet, the relevance and interdependence of these changes for loss of cell–cell adhesion and blistering is poorly understood. In live-cell imaging studies, loss of the keratin network at the cell periphery was detectable starting after 60 min of incubation with immunoglobulin G fractions of PV patients (PV-IgG). These rapid changes correlated with loss of cell–cell adhesion detected by dispase-based dissociation assays and were followed by a condensation of keratin filaments into thick bundles after several hours. Dsg3 internalization started at 90 min of PV-IgG treatment, thus following the early keratin changes. By inhibiting casein kinase 1 (CK-1), we provoked keratin alterations resembling the effects of PV-IgG. Although CK-1-induced loss of peripheral keratin network correlated with loss of cell cohesion and Dsg3 clustering in the membrane, it was not sufficient to trigger the internalization of Dsg3. However, additional incubation with PV-IgG was effective to promote Dsg3 loss at the membrane, indicating that Dsg3 internalization is independent from keratin alterations. *Vice versa*, inhibiting Dsg3 internalization did not prevent PV-IgG-induced keratin retraction and only partially rescued cell cohesion. Together, keratin changes appear very early after autoantibody binding and temporally overlap with loss of cell cohesion. These early alterations appear to be distinct from Dsg3 internalization, suggesting a crucial role for initial loss of cell cohesion in PV.

## Introduction

*Pemphigus vulgaris* (PV) is a severe autoimmune disease affecting the skin and mucous membranes (1). The disease is caused by autoantibodies developing against the transmembrane, cadherin-type cell adhesion molecules desmoglein (Dsg)3 and Dsg1, leading to loss of cell–cell adhesion. This results in blisters predominantly in the mucosa of the oral cavity and the epidermis. Together with desmocollins, Dsgs build up the core of desmosomes, cell–cell adhesion structures abundant in tissues exposed to high degrees of mechanical stress (2). In desmosomes, the transmembrane adhesion molecules form clusters in the membrane and bind extracellularly to their counterparts in the membrane of opposing cells. Intracellularly, they are connected to the intermediate filament network through the linker molecules plakoglobin (Pg), plakophilins (Pkp), and desmoplakin (Dp). This arrangement represents a mechanically stable yet tunable meshwork stabilizing entire tissues (3). The mechanisms leading to loss of cell cohesion in PV are complex as autoantibodies interfere with turnover of desmosomal molecules and desmosome-associated proteins (4, 5). The internalization and depletion of Dsg3 together with other desmosomal components as well as alterations of the keratin intermediate filament (KIF) network are two hallmarks of the disease detectable in biopsies of patient skin and reproduced in disease models (6).

Dsg3 membrane depletion is thought to occur on two levels (4, 5): (i) molecules already transported to the membrane but not yet incorporated into desmosomes are endocytosed leading to interference with desmosome assembly and (ii) existing desmosomes are disassembled and desmosomal molecules or even “half desmosomes” are internalized. The altered turnover of desmosomal molecules is connected to a variety of signaling events in response to autoantibody binding and depends on sufficient lipid rafts. Desmosomal molecules are located in these lipid-enriched membrane domains and both the assembly and disassembly of desmosomes are disturbed upon application of cholesterol-depleting agents (7, 8). Furthermore, p38MAPK, a central molecule deregulated in pemphigus, was shown to be essential for Dsg3 internalization (9) as well as for the KIF network alterations in response to PV-IgG treatment (10, 11).

Keratin intermediate filaments are not static structures but are continuously remodeled to adapt to environmental cues (3, 12). KIFs are nucleated in the cell periphery, elongated and transported toward the nucleus, and disassembled in a perinuclear area to allow reassembly in the cell cortex (13). With regard to turnover rates, it was suggested that a dynamic, quickly changing pool can be distinguished from a stable pool of KIFs inserting in the desmosomal or hemi-desmosomal plaque (14). KIF network dynamics are highly regulated by posttranscriptional modifications, especially phosphorylations (12, 15). *Vice versa*, KIFs regulate the activity of kinases at least in part through scaffolding functions. As an example, KIFs sequester PKCα through the adapter protein RACK1 and stabilize desmosomes by suppressing PKCα activity (16), which was also shown to be disturbed in response to pemphigus autoantibodies (17). Recently, it was demonstrated that casein kinase 1α (CK-1α) localizes to KIFs through FAMH83, which is important for KIF bundling and desmosome turnover (18, 19).

The changes of the KIF network in pemphigus, summarized as “keratin retraction,” are incompletely understood. Early ultrastructural work in patient biopsies demonstrated reduced amounts of KIFs in the cell periphery (20). KIFs condensate to thicker bundles clustering in the perinuclear area and lose contact to the desmosomal plaque. However, it is unclear whether this phenomenon and internalization of desmosomal molecules in response to autoantibodies are linked. Furthermore, it is unknown whether KIF alterations are the cause or rather the consequence of Dsg3 depletion and loss of cell cohesion in PV.

To address these questions, we applied live imaging approaches and biochemical assays to define the temporal relationship between Dsg3 internalization and keratin retraction and to determine how these changes of the cytoskeleton contribute to loss of cell adhesion in PV.

## Materials and Methods

### Cell Culture, Test Reagents, and Constructs

The immortalized human keratinocyte cell line HaCaT and HaCaT cells stably expressing human cytokeratin5 (CK5) fused to Yellow Fluorescent Protein (YFP, kind gift of Reinhard Windoffer and Nicole Schwarz, Institute of Molecular and Cellular Anatomy, RWTH Aachen University) were cultured in Dulbecco’s Modified Eagle Medium supplemented with 10% FCS (Biochrom, Berlin, Germany), 50 U/ml penicillin and 50 U/ml streptomycin (both AppliChem, Darmstadt, Germany), and 0.5 mg/ml G418 in the case of HaCaT-CK5-YFP for selection. Cells were grown in a humidified atmosphere containing 5% CO_2_ at 37°C. Cells were used 24 h after reaching confluency. Medium was changed the day before experiments were performed. Incubations were carried out for the indicated period of time. Casein kinase 1 (CK-1) inhibitor D4476 (Abcam, Cambridge, UK) was used at a concentration of 100 µM. Methyl-β-cyclodextrin (Sigma Aldrich, Munich, Germany), referred to as β-MCD, was applied in a concentration of 1 mM.

pDEST-mDsg3-mCherry-N1 was constructed using the Gateway recombinational cloning system (ThermoFisher, Waltham, MA, USA). In brief, the full-length nucleotide sequence encoding mouse Dsg3 was amplified by PCR using primers carrying attB-specific sites. The primers used were as follow: mDsg3-FW, 5′-GGGGACAAGTTTGTACAAAAAAGCAGGCTTCGAAGGAGATAGAACCatgacctgcctcttcc-3′ and mDsg3-Rev, 5′GGGGACCACTTTGTACAAGAAAGCTGGGTCtagatgggaacaggtttc, where the gateway recombination sequences (including attB sites) are present in capital letters and the lower case letters indicate the sequence complementary to the mouse Dsg3 cDNA. The attB-flanked PCR amplicon was inserted into attP-containing pDONR vector (ThermoFisher) and thus the entry clone was generated. Following the manufacturer’s instruction, the insert was subcloned into a destination vector (pDEST-mCherry-N1, Plasmid #31907, Addgene). As a result, mDsg3 was fused to mCherry on its C-terminus.

### Pemphigus Sera and IgG Purification

Pemphigus sera (PV-IgG) were provided by Enno Schmidt (Lübeck Institute of Experimental Dermatology, University of Lübeck Germany). ELISA titers were as follows: PV1-IgG: anti-Dsg3: 181.44, anti-Dsg1 212.27; PV2-IgG: anti-Dsg3: 206.21, and anti-Dsg1: 182.15. Use of patients’ IgG was approved by the ethics committee (AZ12-178). Additional approval of the study was not required according to the local and national guidelines. Both PV-IgG and an IgG fraction pooled from three different healthy donators (Control-IgG) were purified as described previously (21). In brief, immunoglobulin fractions were extracted from sera through immobilization to protein A agarose (ThermoFisher) in purification columns for 3 h at room temperature. After centrifugation, the serum was removed and the agarose was washed with phosphate-buffered saline (PBS). After elution of the antibodies by sodium citrate buffer (20 mM, pH 2.4) and neutralization with Na_2_CO_3_, a filter unit (Amicon Ultra–4, 100k; Merck Millipore, Darmstadt, Germany) allowed concentration of the IgG fraction at 19,000 *g* for 20 min. IgG was stored in PBS and used in a concentration of 250 µg/ml. AK23, a monoclonal antibody derived from a PV mouse model (Biozol, Eching, Germany) was used in a concentration of 75 µg/ml.

### Immunostaining

HaCaT cells stably expressing CK5-YFP were grown on glass cover slips and fixed with 2% formalin in PBS (freshly prepared from paraformaldehyde) for 10 min at room temperature before being treated with 0.1% Triton X-100 for 5 min to guarantee permeabilization. Subsequently, cells were blocked with 1% normal goat serum and 3% BSA in PBS for 45 min. Cells were incubated with an anti-Dsg3 antibody (clone 5G11, sc-53487, Santa Cruz Biotechnology, Heidelberg, Germany) at 4°C overnight. A Cy3-conjugated secondary goat-anti-mouse antibody (Dianova, Hamburg, Germany) was applied for 1 h at room temperature. 1.5% *N*-propyl gallate was used as an antifading compound to embed the glass dish with cultured cells on glass slides. Images were acquired using a Leica SP5 confocal microscope with a 63× NA 1.4 PL APO objective. Confocal microscopy was performed using lasers with 514 and 543 nm wavelengths for excitation. Analysis was performed using ImageJ (www.nih.gov). The straight bar tool was used to plot intensity profiles of regions of interest. A straight bar of 15 µm length and 20 px width was placed perpendicularly over the cell border of two adjacent cells. Dsg3 was used to indicate the cell border. The position of the bar was not altered for plotting the intensity profile of the respective CK5 image. Resulting intensity profiles indicating the distribution of the protein of interest were compiled in Excel (Microsoft, Redmond, WA, USA) and subsequently normalized to the baseline.

### Western Blot and Lysates

Cells were washed with PBS and subsequently lysed with SDS-lysis buffer (25 mmol/l HEPES, 2 mmol/l EDTA, 25 mmol/l NaF and 1% SDS, pH 7.4) followed by sonification. Protein amount was determined using the BCA method (ThermoFisher, USA). A mixture of lysate and Laemmli buffer containing 50 mM dithiothreitol was prepared and 10 µg of protein were loaded on a gel for electrophoresis. Electrophoresis and western blotting were carried out according to standard procedures. Membranes were blocked in either 5% skim milk powder dissolved in Tris-buffered-saline containing 0.05% tween (TBS-T) or 5% BSA in TBS-T at room temperature for 1 h. The following antibodies were applied in BSA in TBS-T at 4°C overnight: Dsg3 pAb (ELA-EAP3816-120, Biozol), Desmoplakin I/II pAb (H-300) (sc-33555, Santa Cruz), CK14 mAB (LL002) (ab7800, Abcam, Cambridge, UK), GAPDH mAb (0411) (sc-47724, Santa Cruz), phospho-p38 MAPK pAb (Thr180/Tyr182, D3F9) (#4511, Cell Signaling Cambridge, UK), and p38 MAPK pAb (#9212, Cell Signaling). HRP-coupled goat-anti-mouse Ab or goat-anti-rabbit Ab (both Dianova) were applied as secondary antibodies at room temperature. Membranes were developed using the ECL system (GE Healthcare, Munich, Germany). Western blots were analyzed by measuring the integrated density of bands after background subtraction using ImageJ.

### Triton X-100 Protein Fractionation

HaCaT-CK5 cells were put on ice and washed with ice-cold PBS. Extraction buffer (0.5% Triton X-100, 50 mmol/l MES, 25 mmol/l EGTA, 5 mmol/l MgCl_2_) containing 0.1% leupeptin, aprotinin, and pepstatin as well as 1% phenylmethylsulfonyl fluoride was applied for 10 min under gentle shaking on ice followed by scraping to retrieve lysates. Centrifugation at 19,000 *g* for 10 min at 4°C enabled the separation of the cytoskeletal insoluble fraction from the triton-soluble non-cytoskeletal-bound fraction. The Triton X-100 soluble fraction was harvested and processed separately for blotting. The obtained pellet representing the insoluble fraction was suspended in SDS-lysis buffer (25 mmol/l HEPES, 2 mmol/l EDTA, 25 mmol/l NaF, and 1% SDS, pH 7.4) and sonicated. Protein levels of both fractions were measured using the BCA method (ThermoFisher). 5 or 10 µg of each fraction were mixed with Laemmli buffer and subjected to Western blotting.

### Biotinylation Assay

After incubation, HaCaT-CK5 cells were put on ice and thoroughly washed with HbSS. This step was followed by a 1 h incubation with 0.25 mM membrane-impermeable EZ-Link Sulfo-NHS-Biotin (ThermoFisher). To remove excess biotin, cells were washed with ice-cold HbSS containing 100 mM Glycin and plain HbSS afterward. Under gentle shaking, cells were incubated with cooled lysis buffer (50 mM NaCl, 10 mM PIPES, 3 mM MgCl_2_, 1% Triton X-100, 1% phenylmethylsulfonyl fluoride, 0.1% of each leupetin, aprotinin, and pepstatin) for 20 min on ice. Lysates were acquired through scraping and subsequently centrifuging at 19,000 *g* for 5 min. Supernatant was retrieved and protein concentrations were measured using the BCA method (ThermoFisher). 250 µg of protein were mixed with 70 µl of NeutrAvidin (HighCapacity)-agarose (ThermoFisher) and put on a rotator overnight at 4°C. The next day, agarose beads were washed five times with cold lysis buffer. Biotinylated-protein attached to the agarose was suspended in 3× Laemmli buffer containing 50 mM dithiotreitol (AppliChem). Lysates were loaded on gels and Western blotting was carried out as described above. Biotin of the biotin-bound fraction was detected using streptavidin-HRP (Cell Signaling), the integrated density of the entire lane of each condition was measured and used as a loading control. Representative sections of the membranes are shown in the figures. The whole lysate was normalized to GAPDH.

### Phos-tag™ Assay

To determine the phosphorylation state of a protein, a Manganese (II)-Phos-tag™ SDS-PAGE (Wako Chemicals GmbH, Steinbach, Germany) was carried out. The Phos-tag component which is incorporated in the gel reduces the migration speed of phosphorylated proteins in the electrophoresis process. The imbalance of migration speed between phosphorylated proteins and their lower or non-phosphorylated counterparts enables the detection of a phosphorylation state. After the indicated treatment of cells with D4476 reagent, HaCaT-CK5 cells were put on ice and washed with prechilled TBS and lysed in Laemmli buffer supplemented with 0.1% of each leupeptin, pepstatin, and aprotinin, 1% of phenylmethylsulfonyl fluoride, phosphatase inhibitor (Roche), and 50 mM dithiotreitol. Manganese (II)-Phos-tag™ SDS-PAGE was performed according to the manufacturer’s instructions. For electrophoresis 6% polyacrylamide gels were freshly prepared containing either 0 mM (control gel) or 30 mM Phos-tag™ ALL-107 (Wako Chemicals). Lysates were sonicated and loaded on the gels. Mn^2+^ was removed from the gels by washing twice for 10 min in transfer buffer containing 30 mM EDTA. A third washing step followed in transfer buffer without EDTA equally for 10 min. Proteins were transferred to a PVDF-membrane (Bio-Rad Laboratories, Munich, Germany). Antibodies were applied as detailed in the Western blot section.

### Dispase-Based Dissociation Assay

HaCaT keratinocytes were seeded in duplicate for each condition and grown for 24 h after reaching confluency. Cells were washed with prewarmed PBS and subsequently incubated with HbSS containing Dispase II (>2.4 U/ml; Sigma Aldrich) for 20 min at 37°C in order to detach the cell monolayer from the well bottom. Dispase II solution was replaced by HbSS. A defined sheer stress was applied to the cell sheets by pipetting the monolayer 10 times using an electrical 1 ml pipet. Increased numbers of fragments, counted under a binocular microscope, compared to control conditions indicated the loss of intercellular adhesion. For better display of the fragments, 10 µM thiazolyl blue tetrazolium bromide (MTT) (SigmaAldrich) were added to the vials for 20 min to obtain staining of viable cells.

### Live Cell Imaging

HaCaT-CK5 cells were seeded in μ-slide eight-well imaging chambers (Ibidi, Martinsried, Germany) and grown to confluency. Dulbecco’s Modified Eagle Medium with supplements was replaced with Dulbecco’s Modified Eagle Medium without phenol red. Transfection with pDest-mDsg3-mCherry for double transfection experiments was carried out 3 days after cell seeding at a confluency of 90% with Lipofectamine LTX with Plus Reagent (ThermoFisher). The transfection was performed according to the manufacturer’s protocol, incubating the cells with a final concentration of 3 µg plasmid DNA, 3 µl Plus-Reagent and 5 µl of LTX-Reagent per milliliter for 4 h. Experiments were carried out 24 h after transfection. Live Cell Imaging experiments were performed using a Leica SP5 confocal microscope with a 63× NA 1.2 PL APO water or a 63× NA 1.4 PL APO oil objective. An incubator housing (OKOLAB, Pozzuoli, Italy) guaranteed a constant humidified atmosphere at 37°C with 5% CO_2_.

Untransfected HaCaT CK5-YFP cells were imaged with a 20-mW argon laser at 514 nm wavelength and 3% laser power. A stack was created covering the entire z-dimension of the monolayer in 0.5 µm steps and 512 × 512 pixel stacks were acquired every 30 s. Z-stacks of HaCaT CK5-YFP cells transfected with Dsg3-mCherry were generated by sequential imaging using the 514 nm argon laser and a 10-mW 543 nm laser line at 20% power. Images were acquired every 2 min.

### Live Cell Imaging Analysis

All stacks were deconvolved using the software Huygens Essentials (Scientific Volume Imaging, Hilversum, The Netherlands) at a signal to noise ratio of 10 and a maximum of 30 iterations. Further analysis of otherwise raw image data was performed with ImageJ, unless specified otherwise.

To analyze the number of keratin bridges of adjacent cells, a maximum intensity projection of all layers was used. KIFs “bridging” two neighboring cells were manually counted and put in relation to the length of the respective cell border area. For intensity measurement in the cell periphery, a projection of layers covering the lower 4 µm of the cell was applied. The mesh size of KIFs differs from the cell periphery toward the nucleus (22), a phenomenon which was also evident in HaCaTs expressing CK5-YFP. Following this indication, the mean intensity of the cell periphery with a large mesh size and the mean intensity of the dense perinuclear KIF network were independently analyzed using the drawing tool of ImageJ. To quantify loss of keratins in the cell periphery, the peripheral KIF intensity values were divided by the perinuclear intensity values.

Keratin thickening was quantified in maximum intensity projections. Intensity levels of independent experiments were adjusted to the same baseline and further processed with the software CellProfiler (23). In a first step, the tubeness filter was applied. A threshold of 0.2 was defined through empiric trials and applied to the images in a next step. Bridging and cleaning filters were applied to close small gaps between KIF bundles or to remove one-pixel error signals, respectively. Resulting images depicted high intensity KIF signals as white, corresponding to thickened keratin bundles. The mean intensity of these images was used as measure for KIF thickening.

The intensity of Dsg3 in the membrane was analyzed using a projection of three layers (1.5 µm) located above and below the plane with the largest nuclear diameter. Only cells with clear membrane localization were included for imaging. The mean intensity of a 2 µm wide region of interest containing the entire cell circumference was compared with the intensity of the remaining cytoplasm (excluding the nucleus region devoid of any signals). We used this index to detect an intensity switch from the cell border into the cytoplasm as an indicator for Dsg3 internalization.

To determine Dsg3 membrane clustering, a bar of 10 µm length and 10 px width was drawn over the Dsg3 signal at the membrane and the intensity profile was plotted every 12 min at the same region. Clustering was indicated by intensity peaks in the profile developing over time. These were quantified as follows: First, the average intensity value of the initial 0 min profile was calculated. This value was multiplied by 1.5 and used as threshold for the subsequent time points. To detect newly occurring peaks in the profiles, we calculated the area under the curve above this threshold.

### Data Processing and Statistics

Photoshop CC 2017 (Adobe Systems, San Jose, CA, USA) was used for image processing and compilation. We used Excel (Microsoft, Redmond, WA, USA) for data analysis. Statistical significance was determined using paired Student’s *t*-test for two-group comparisons in Excel or one-way ANOVA followed by Bonferroni correction using Graphpad Prism (Graphpad Software, LaJolla, CA, USA) for comparison of more than two groups. Significance was presumed with *p* < 0.05. Data shown are mean ± SEM.

## Results

### Pemphigus Autoantibodies Induced Dsg3 Depletion and Keratin Retraction *In Vitro*

**Figure 1:**
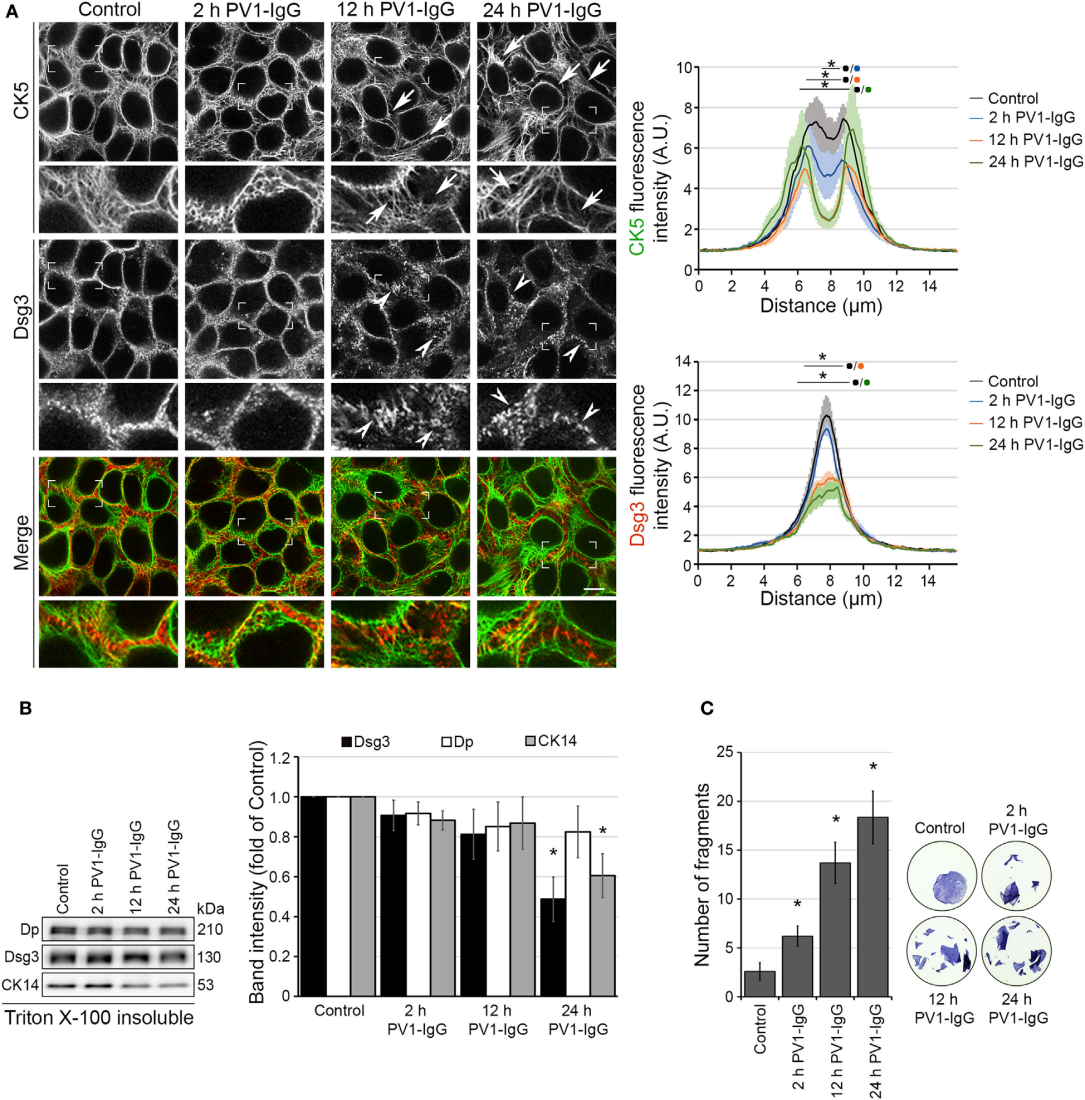
Structural changes of human keratinocytes in response to pemphigus vulgaris (PV) antibody exposure. HaCaT keratinocytes expressing cytokeratin5 (CK5)-YPF (HaCaT-CK5) were incubated with PV1-IgG for 2, 12, or 24 h. Images shown are representatives of >3 independent experiments. **(A)** Desmoglein (Dsg)3 staining and CK5 expression in response to the antibody binding. Loss of keratin filaments in the cell periphery is marked by arrows and Dsg3 alterations by arrowheads. Comparison of fluorescence profiles on a 15 µm line perpendicularly to the membrane of two adjacent cells (*n* = 75 cells from three independent experiments, *p < 0.05 vs. control). Bar represents 10 µm. **(B)** Triton X-100 insoluble fraction, representing the cytoskeletal-bound fraction, of HaCaT-CK5 lysates after incubation with PV1-IgG for the indicated period of time (*n* = 6). Densitometric analysis of structure proteins shown as fold of control (*n* = 6, *p < 0.05 vs. control). **(C)** Dispase-based dissociation assays in HaCaT-CK5 keratinocytes after PV1-IgG treatment (*n* = 5, *p < 0.05 vs. control). Representative images of cell sheets after applied sheer stress stained with 10 µM MTT for better visibility.

Initially, we characterized changes in Dsg3 and keratin distribution under static conditions in cultured human keratinocytes. HaCaT keratinocytes stably expressing YFP-tagged CK5 were incubated with PV1-IgG for 2, 12, and 24 h (Figures 1A,B). After 12 h and even more so after 24 h incubation, the keratin cytoskeleton appeared retracted from the cell periphery (arrows) and Dsg3 clustering together with reduced localization along the cell border was visible. In line with previous data (24), Dsg3 was linearized in arrays perpendicular to the cell border and intracellular Dsg3 clusters (arrowheads) reminiscent of internalization were present. At 2 h, the distribution of Dsg3 showed subtle changes and a beginning reduction of keratin intensity at cell borders was detectable. At 24 h, the amount of CK5 and Dsg3 was reduced in the cytoskeletal (Triton X-100 insoluble) pool (Figure 1B). This indicates disassembly of the KIF network after longer periods of PV-IgG treatment.

Despite only minor detectable changes by immunostaining at 2 h of PV1-IgG incubation, cell–cell adhesion was compromised as revealed by dispase-based dissociation assays (Figure 1C). In agreement with the structural changes, cell cohesion was more strongly impaired at later time points. Together, the cell line applied here demonstrates the typical hallmarks of the disease also evident in keratinocytes from pemphigus patient skin (25).

**Figure 2:**
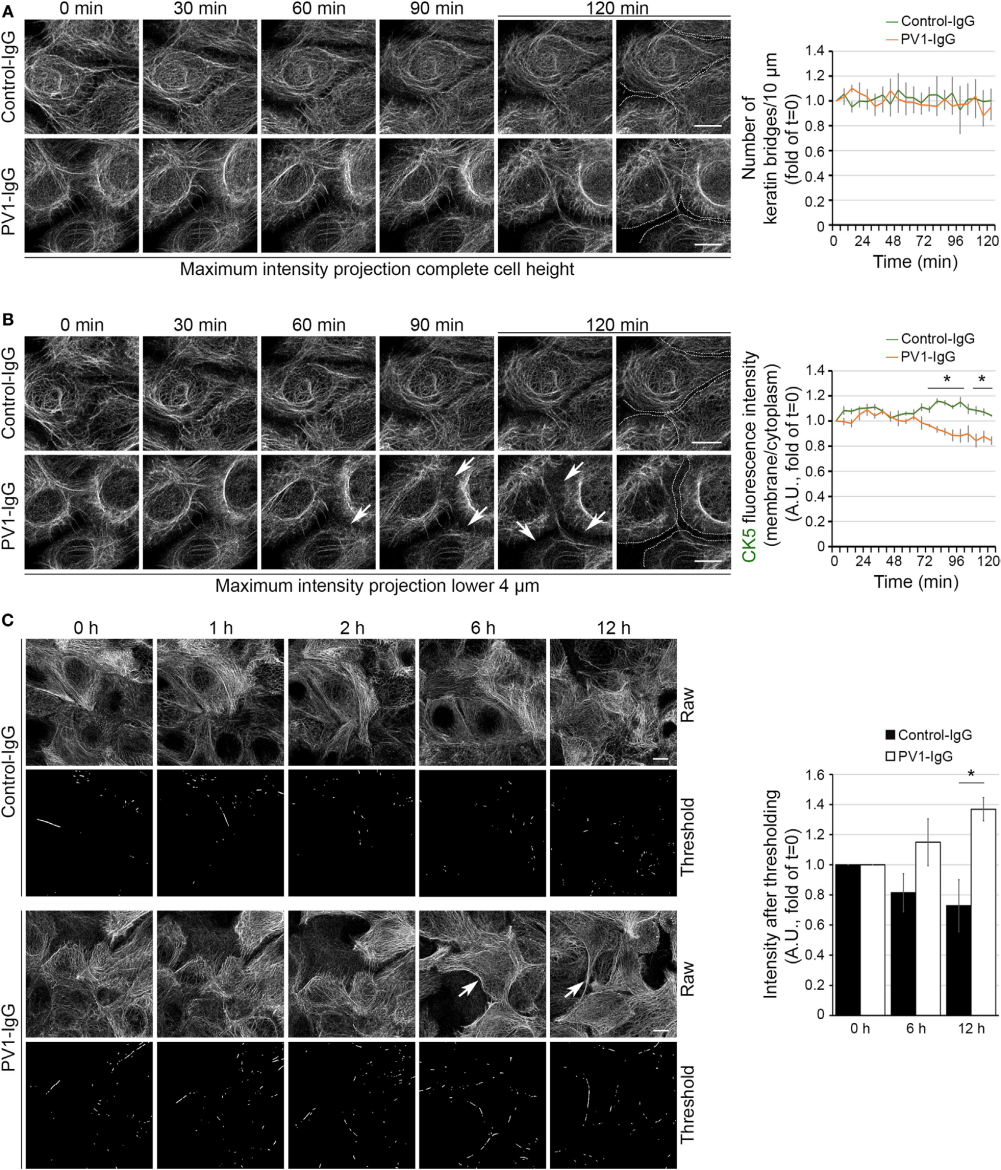
Time course of keratin changes after antibody binding. Representative image sequence of high resolution three-dimensional confocal time-lapse microscopy as a maximum intensity projection of the complete HaCaT-CK5 cell height **(A)**. Images were acquired in 30-s intervals and number of keratin bridges was assessed every 6 min after PV1-IgG addition (*n* = 8–10 cells from three to four independent experiments, *p < 0.05 vs. Control-IgG at respective time point). **(B)** In a maximum intensity projection containing the lower 4 µm of the cell body, the ratio of fluorescence intensity in the cell periphery to fluorescence intensity in the cytoplasm was analyzed every 6 min. Arrows mark loss of fluorescence intensity in the cell periphery. Dashed lines indicate the cell border area and were drawn for better visualization (*n* = 8–10 cells from three to four independent experiments, *p < 0.05 vs. Control-IgG at respective time point). **(C)** Keratin bundling (arrows) was caused by PV1-IgG exposure. Bundling was visualized by putting a threshold on raw data and measuring the remaining intensity (*n* = 3–4, *p < 0.05 vs. Control-IgG at respective time point). Bars represent 10 µm.

The time course of KIF changes in response to PV-IgG is largely unknown. Using atomic force microscopy imaging, an altered cytoskeletal meshwork in living keratinocytes within the first 2 h was demonstrated recently (26). Because KIF and Dsg3 changes started around 2 h after autoantibody addition in static experiments, we next performed live cell imaging in the first 2 h using HaCaT keratinocytes stably expressing CK5-YFP to detect potential discrete changes in the same cells. In this approach, we carried out high resolution three-dimensional confocal time-lapse microscopy. Z-stacks spanning the entire cell height were acquired in 30-s intervals and changes in response to PV1-IgG were analyzed in maximum intensity projections. We first investigated the number of filaments running perpendicular to the cell membrane (Figure 2A). Interestingly, these structures known to be relatively stable did not change within 2 h in absence or presence of PV-IgG. To detect potential effects which might be concealed in maximum intensity projections, we separately analyzed the bottom 4 µm of the keratinocyte monolayer, in which the KIF network was most volatile (Figure 2B; see Video S1 in Supplementary Material). Indeed, these image series showed a significant loss of keratin fluorescence in the cell periphery starting around 60 min of PV-IgG incubation. At these early time points, the overall extent of the observed changes was rather moderate but clearly detectable by analyzing the signal intensities in the cell periphery. To evaluate the distribution in longer time courses, we performed overview experiments in lower magnification for up to 12 h with a reduced temporal resolution. Interestingly, KIFs condensed into thicker bundles which became visible after around 6 h of PV1-IgG treatment and progressed further on (Figure 2C). For analysis, a thresholding step was applied and the amount of signal exceeding this threshold was analyzed (see Materials and Methods).

Together, the live cell imaging experiments suggest a biphasic impact of autoantibody binding on keratin filament distribution. Initially, a pool located in the cell periphery and potentially being responsible for KIF assembly is reduced which is followed by condensation of more stable filaments anchoring desmosomes.

### Keratin Changes Paralleled Dsg3 Clustering and Preceded Dsg3 Internalization

**Figure 3:**
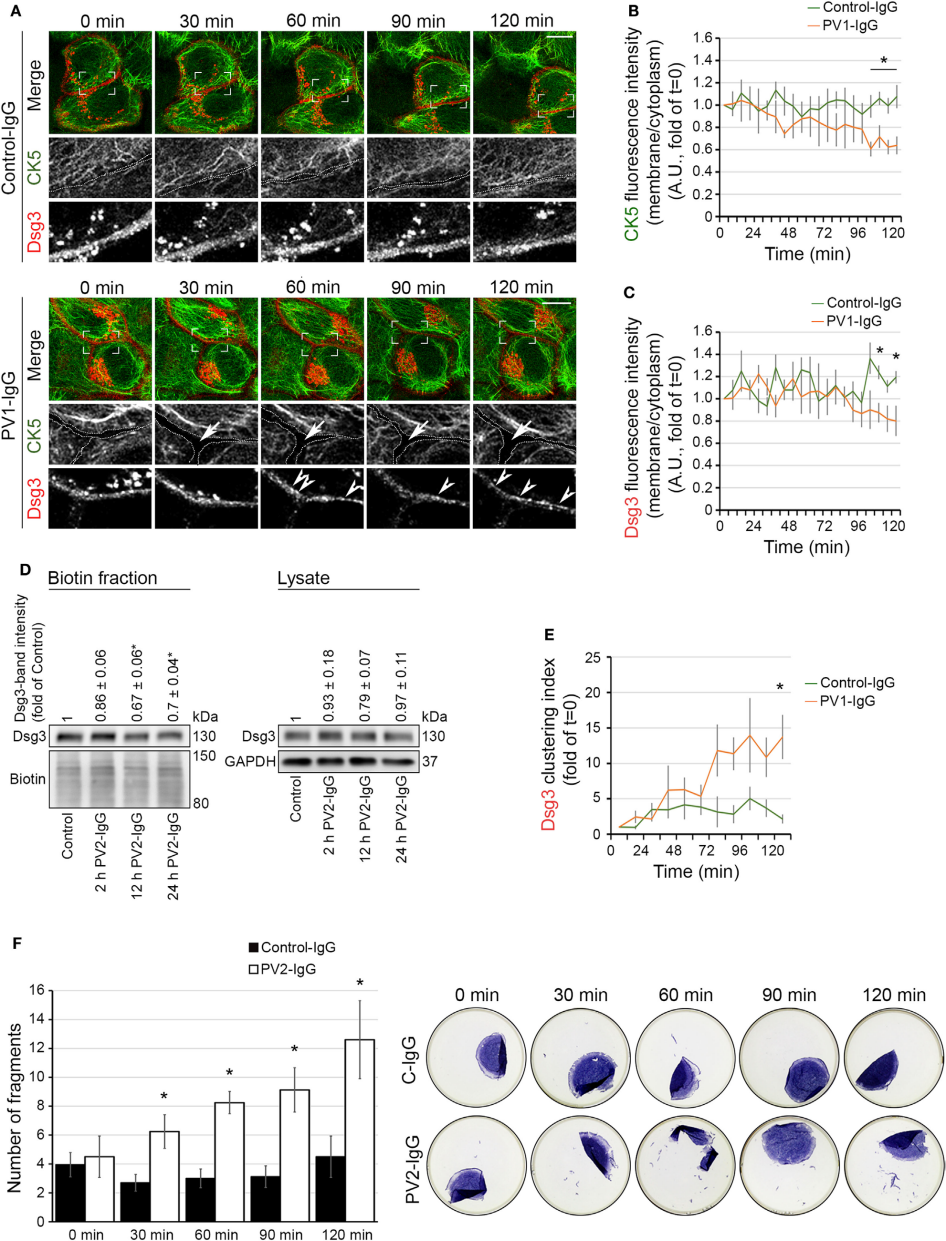
Keratin changes and Dsg3 clustering occurred in parallel and preceded Dsg3 internalization. HaCaT keratinocytes expressing CK5-YFP were transfected with Dsg3-mCherry. In three-dimensional confocal time-lapse microscopy experiments, cells were incubated with either PV1-IgG or Control-IgG and imaged every 2 min for 120 min. Dashed lines indicate cell border areas **(A)**. Ratio of CK5 fluorescence intensity and in the cell periphery vs. intensity in the cytoplasm was assessed **(B)** (*n* = 5–6 cells, each from an independent experiment, *p < 0.05 vs. Control-IgG at respective time point). Ratio of Dsg3 distribution in the membrane vs. the cytoplasm **(C)** (*n* = 5–6, *p < 0.05 vs. Control-IgG). Streptavidin pulldown of biotinylated membrane Dsg3 after 2, 12, and 24 h of PV2-IgG incubation and densitometric analysis of protein levels (*n* = 3–4, *p < 0.05 vs. control) **(D)**. Dsg3 clustering was evaluated with intensity profile plotting linearly along the membrane (*n* = 5–6, *p < 0.05 vs. Control-IgG at respective time point) **(E)**. Loss of cell adhesion caused by incubation with PV2-IgG was measured in parallel to structural changes at early time points by dispase-based dissociation assay **(F)** (*n* = 4–5, *p < 0.05 vs. respective Control-IgG incubation).

Next, we further investigated the temporal relationship between keratin alterations and Dsg3 internalization. We generated a Dsg3 construct C-terminally fused with mCherry and used it for transfections of HaCaT cells stably expressing CK5-YFP. Similar to the experiments without double transfection, a reduction of the keratin network in the cell periphery became evident starting at 60 min of PV1-IgG incubation, indicating that Dsg3 overexpression did not alter keratin dynamics (Figures 3A,B; see Video S1 in Supplementary Material). Dsg3 signals at cell borders were stable for 90 min of PV1-IgG incubation and were starting to become reduced afterward as demonstrated by analysis of membrane intensities (Figures 3A,C). Thus, the amount of Dsg3 in the membrane appeared to be reduced later than the CK5 changes. The exogenous CK5 expression was not protective with regard to Dsg3 internalization, as wild-type HaCaT cells transfected with Dsg3-GFP displayed similar results (Figure S1 in Supplementary Material). To support these imaging data, we performed cell surface biotinylation assays to biochemically determine the extent of Dsg3 membrane depletion (Figure 3D). In agreement with live imaging data, only a minor reduction of Dsg3 membrane levels was detectable after 2 h, whereas depletion from the membrane was pronounced after 12 and 24 h of PV2-IgG incubation, respectively. Stable Dsg3 membrane levels do not rule out alterations in the distribution of the molecules. Clustering of Dsg3 molecules within the membrane in response to PV-IgG was shown to precede internalization (24). We thus analyzed Dsg3 clustering in live cell experiments which was detectable first after 60 min of PV-IgG incubation and proceeding over time (Figures 3A,E, arrowheads). In parallel to these experiments, we closely monitored cell cohesion by dispase-based dissociation assays (Figure 3F). Compared to the gross disruption of the monolayer typically evident at 24 h of incubation, at these early time points the monolayer fragmentation was mainly detectable at the periphery presumably because cells are slightly less dense in this region. Compared to Control-IgG, fragmentation and thus loss of cell–cell adhesion was significantly increased beginning at 30 min of PV2-IgG incubation and increased further on.

These results demonstrate that changes in KIF distribution par-allel loss of cell cohesion and Dsg3 clustering but precede a morphologically and biochemically detectable Dsg3 internalization.

### CK-1 Inhibition Altered Keratin Distribution Similar to PV-IgG

The observation that KIF changes correspond to Dsg3 clustering but precede Dsg3 depletion from the membrane is suggestive of a keratin-dependent regulation of Dsg3 turnover. To test this hypothesis, an approach to induce keratin retraction independent from autoantibody binding to Dsg3 is required. Recently, CK-1α was identified to mediate keratin cytoskeleton organization in a FAM83H-dependent manner (18). CK-1α regulates the filamentous state of keratin filaments and its inhibition promotes keratin bundling in proximity to the nucleus. The phenotype of keratin reorganization under CK-1α inhibition closely matched the one that is observable in PV.

**Figure 4:**
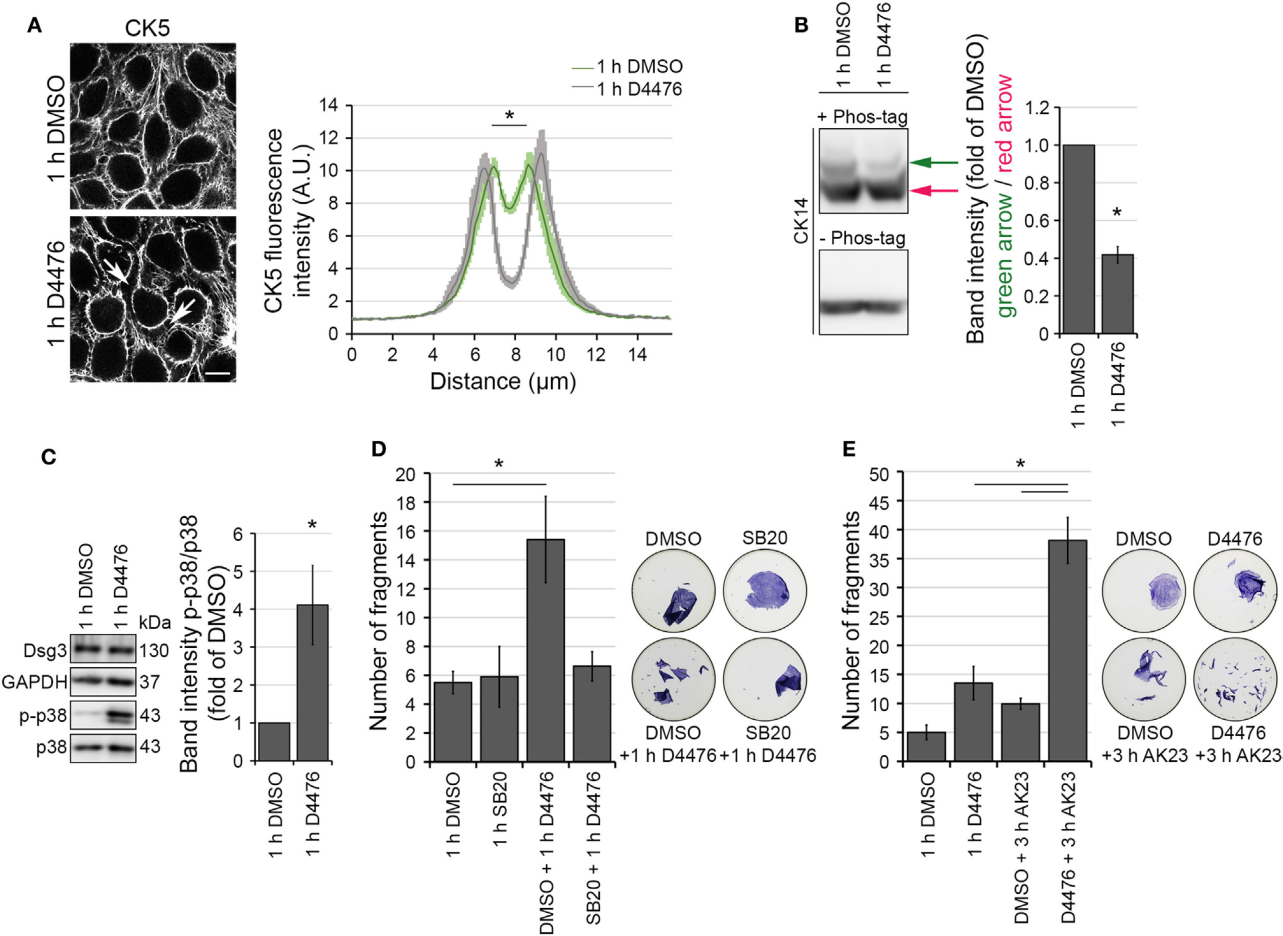
Casein kinase 1 inhibition provoked keratin alterations resembling the PV-IgG-induced phenotype. HaCaT-CK5 cells were incubated with CK-1 inhibitor D4476 for 1 h. The resulting keratin retraction (arrows) was measured with a bar of 15 µm length perpendicular to the cell border of two adjacent cells **(A)** (*n* = 75–100 cells from three to four independent experiments, *p < 0.05 vs. 1 h DMSO). Bar is 10 µm. **(B)** Phos-tag™ was incorporated in a 6% Western blot gel (+ Phos-tag) to detect the phosophorylation status of CK14 in HaCaT-CK5 cells. Control gel without Phos-tag (– Phos-tag) excluded protein fragmentation. Band intensity of both phosphorylation sites was measured and the first phosphorylation site (green arrow) was divided by the basal phosphorylation site (red arrow) (*n* = 4, *p < 0.05 vs. 1 h DMSO). **(C)** SDS lysates were generated from cells treated with 1 h D4476 or 1 h DMSO and p38MAPK phosphorylation was evaluated. Densitometric results were normalized to total p38 levels as a loading control (*n* = 6, *p < 0.05 vs. 1 h DMSO) Cell adhesion was quantified in HaCaT keratinocytes by dispase-based dissociation assays. Pre-incubation with SB203580 (SB20) as a specific inhibitor of p38MAPK or DMSO as a control for 1 h was followed by D4476 incubation for 1 h (*n* = 4–5, *p < 0.05) **(D)**. Pre-incubation with D4476 or DMSO for 1 h followed by 3 h incubation with AK23, a pathogenic monoclonal desmoglein (Dsg)3 antibody **(E)** (*n* = 4, *p < 0.05). Remaining cell sheets and fragments were stained with 10 µM MTT for visualization.

We visualized the effect of the CK-1 inhibitor D4476 on HaCaT cells expressing CK5-YFP. Inhibition of CK-1 induced peripheral keratin filament loss after 1 h, resembling PV-IgG-induced keratin retraction (Figure 4A). In line with a regulation of KIFs by CK-1, D4476-induced dephosphorylation of CK14 as indicated by Phos-Tag experiments (Figure 4B). Keratin retraction in response to autoantibody binding in PV is associated with a rapid increase of p38MAPK signaling. Inhibition of p38MAPK prevents keratin cytoskeleton rearrangement and loss of cell cohesion (10). Also, the PV-IgG fraction applied here induced p38MAPK activation (Figure S1B in Supplementary Material; See also Figure 6C). Similar to the effects of PV-IgG, phospho-p38MAPK levels were increased in response to D4476 incubation for 1 h compared to control conditions (Figure 4C). Finally, D4476 incubation induced loss of cell cohesion which was prevented by inhibition of p38MAPK through SB203580 (Figure 4D; Figure S1C in Supplementary Material). Nevertheless, reduced cell cohesion in response to D4476 was further impaired by additional application of AK23, a monoclonal anti-Dsg3 autoantibody derived from a pemphigus mouse model (Figure 4E; Figure S1D in Supplementary Material). Taken together, KIF alterations in response to CK-1 inhibition through D4476 resemble PV-IgG-induced keratin retraction.

### Keratin Retraction Did Not Induce Dsg3 Internalization

**Figure 5:**
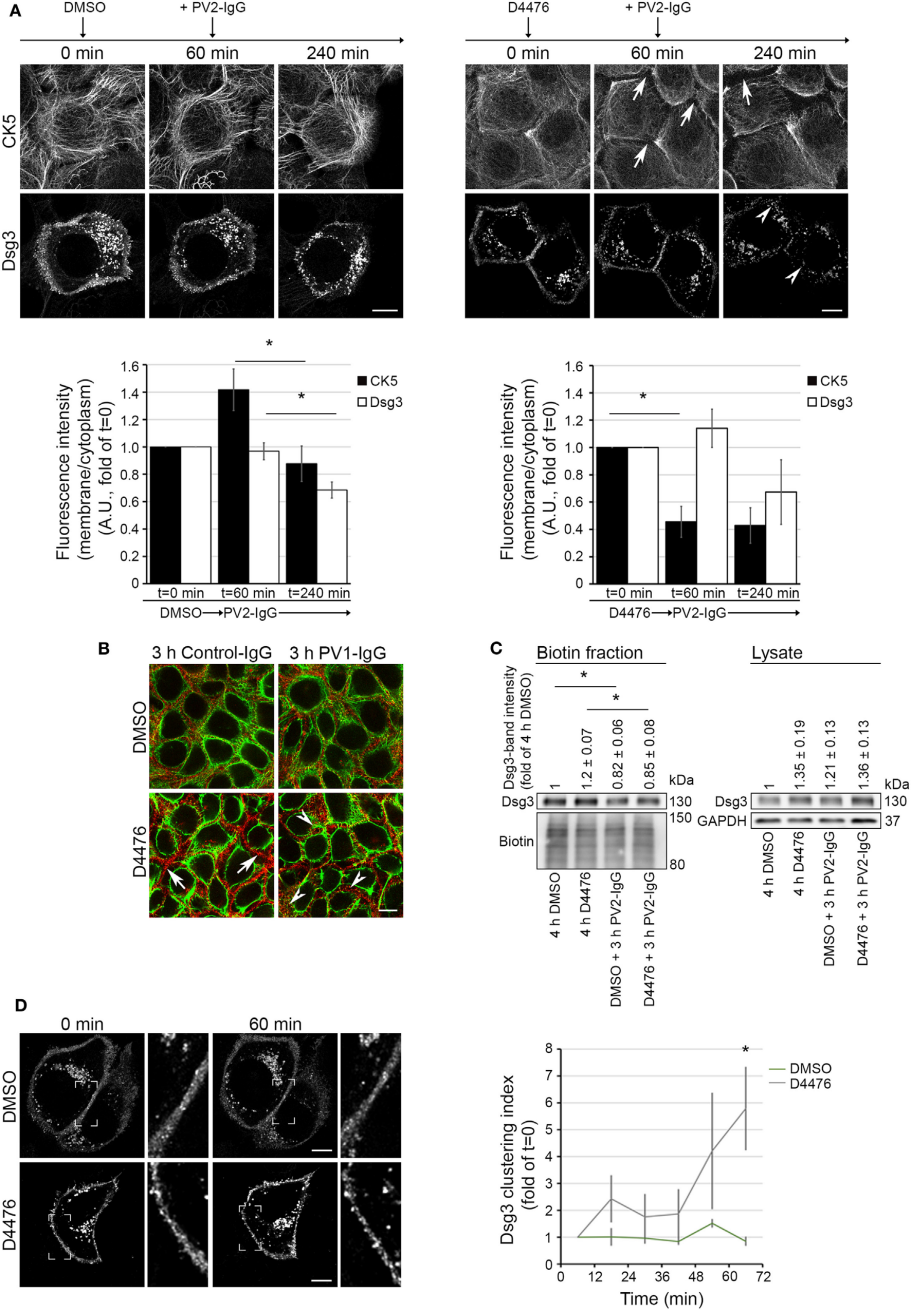
D4476-induced keratin changes did not induce Dsg3 internalization. HaCaT-CK5 keratinocytes were transfected with pDest-mDsg3-mCherry, incubated with D4476 or DMSO and followed up by three-dimensional confocal time-lapse microscopy **(A)**. D4476 caused keratin retraction after 60 min (arrows) but did not lead to internalization of Dsg3. Subsequent addition of PV2-IgG caused Dsg3 membrane depletion (arrowheads). Bar represents 10 µm (*n* = 4 cells from 4 independent experiments, *p < 0.05). **(B)** Immunostaining of HaCaT-CK5 keratinocytes and Dsg3. Keratin changes occurred under D4476 treatment (arrows) and signs of Dsg3 fragmentation and internalization were visible in response to PV1-IgG treatment only (arrowheads). Images shown are representative for three to four independent experiments. Bar represents 10 µm. **(C)** Streptavidin pulldown of biotinylated Dsg3 in HaCaT-CK5 cells incubated with DMSO or D4476 for 4 h as a control and 1 h incubation followed by a 3 h PV2-IgG incubation. Dsg3 membrane levels were densitometrically assessed (*n* = 3–5, *p < 0.05 vs. respective control condition). **(D)** Dsg3-clustering was analyzed in 1 h live cell imaging sequence of DMSO or D4476 incubation. Clustering was detected using a bar of 10 µm applied linearly on the membrane (*n* = 4, *p < 0.05 vs. DMSO at respective time point). Bar represents 10 µm.

To elucidate a possible dependence of keratin retraction on Dsg3 distribution, we applied D4476 in live cell imaging experiments with human keratinocytes expressing CK5-YFP transfected with Dsg3-mCherry. Cells were pre-incubated with either D4476 or DMSO, followed by 3 h of PV2-IgG incubation (see Video S2 in Supplementary Material). Image analysis showed a drastic reduction of keratin cytoskeleton fluorescence in the cell peripherywhen inhibiting CK-1 which did not occur under control conditions with DMSO (Figure 5A, arrows). Interestingly, the retraction of the keratin cytoskeleton was not paralleled by internalization of Dsg3. However, additional incubation with PV2-IgG promoted internalization of Dsg3 when the cytoskeleton was already altered (arrowheads). We further substantiated these findings by im-munostaining under conditions without Dsg3 overexpression (Figure 5B). Similar to the live cell experiments, Dsg3 appeared unaffected under conditions of D4476-mediated keratin retraction. Nevertheless, Dsg3 clustering and intracellular Dsg3 vesicles were found in both conditions after additional incubation with PV1-IgG (arrowheads). In support of these data, streptavidin pull down of biotinylated surface molecules confirmed the absence of Dsg3 depletion from the membrane in D4476-treated cultures which was induced upon additional 3 h incubation with PV2-IgG (Figure 5C).

Interestingly, although overall membrane levels were not altered, the analysis of Dsg3 distribution revealed Dsg3 clustering in the membrane after 60 min of D4476 treatment (Figure 5D). These data show that D4476-mediated KIF redistribution did not induce Dsg3 internalization but contributed to clustering in the membrane. This suggests that Dsg3 clustering and internalization of Dsg3 are distinct events and demonstrate the dependence of Dsg3 clustering on correct distribution of the KIF network. By contrast, Dsg3 internalization appears to be independent from KIF alterations and may be a result of other mechanisms elicited by autoantibody binding to Dsg3.

### Inhibition of Dsg3 Internalization Did Not Prevent Keratin Retraction

**Figure 6:**
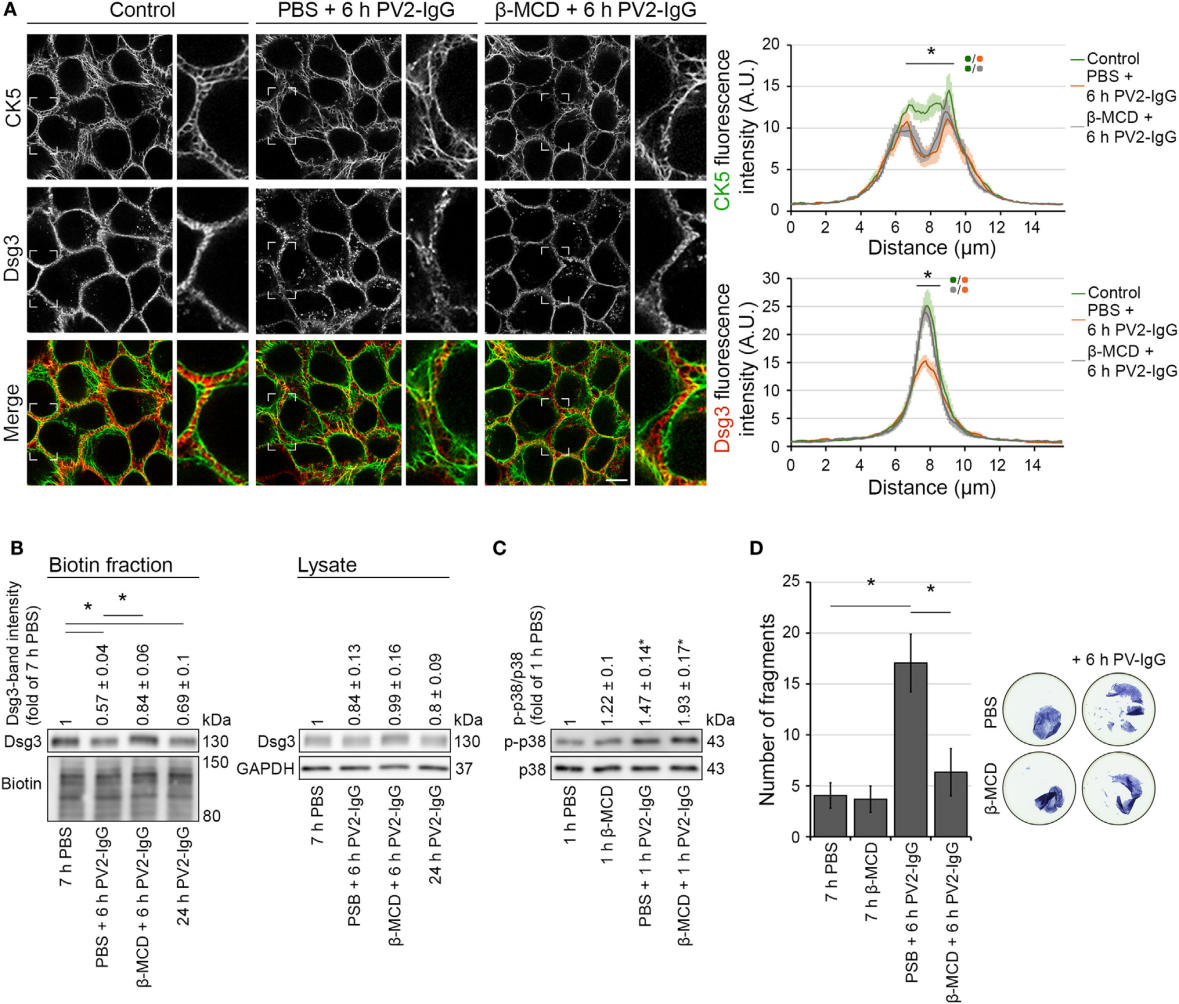
Inhibition of Dsg3 internalization through cholesterol depletion did not prevent PV-IgG-driven keratin alterations. HaCaT-CK5 keratinocytes were pre-incubated with either PBS or β-MCD as a cholesterol-depleting agent for 1 h and then exposed to PV2-IgG for 6 h. Images shown **(A)** are representative for four independent experiments. For analysis a bar of 15 µm was applied perpendicularly over the cell border of two adjacent cells and the fluorescence intensity was plotted for both Dsg3 and CK5 (*n* = 100 cells from four independent experiments; *p < 0.05). Bar represents 10 µm. **(B)** Streptavidin pulldown of biotinylated membrane Dsg3 was performed with HaCaT-CK5 keratinocytes (*n* = 3–5, *p < 0.05). **(C)** Phosphorylation of p38MAPK was determined by densitometry. **(D)** Intercellular adhesion was determined by dispase-based dissociation assays in HaCaT keratinocytes (*n* = 8, *p < 0.05).

Desmosome assembly and disassembly were shown to be dependent on lipid-raft integrity (7, 27), which can be disturbed by the cholesterol-depleting agent methyl-β-cyclodextrin (β-MCD). In this regard, it was shown that disruption of lipid raft formation restrains Dsg3 endocytosis following PV antibody exposure (7). We used β-MCD to investigate the dependence of keratin alterations triggered by PV-IgG on Dsg3 internalization. Immunostaining and biotinylation assays of human keratinocytes expressing CK5-YFP revealed that 1 h pre-incubation with β-MCD largely prevented Dsg3 internalization after autoantibody exposure (Figures 6A,B). However, keratin retraction was still present under these conditions, indicating independency of Dsg3 alterations. Despite the restriction of Dsg3 internalization through lipid raft disruption and in line with a p38MAPK-dependent regulation of the keratin network, p38MAPK activation by PV1-IgG was not abolished (Figure 6C). In dispase-based dissociation assays, loss of cell–cell adhesion caused by PV2-IgG was ameliorated through lipid raft disruption (Figure 6D). Taken together, these results suggest that keratin alterations are independent from Dsg3 endocytosis following PV-IgG incubation. Moreover, both effects apparently contribute to loss of cell cohesion. This suggests different mechanisms driving keratin alterations and Dsg3 internalization.

## Discussion

In the present study, we show a precise time course of Dsg3 endocytosis and keratin retraction in response to PV-IgG, dissecting the temporal relationship of two hallmarks of PV. Our results demonstrate that both mechanisms contribute to loss of cell cohesion but appear to be largely independent of each other. Based on the observation that cell dissociation is correlating with keratin changes before onset of Dsg3 internalization, these results suggest an important role of keratin retraction for loss of cell cohesion and blistering.

### Keratin Alterations Precede Dsg3 Internalization

It is a matter of debate whether KIF alterations in response to pemphigus autoantibodies are secondary to changes of the desmosomes or rather cause and contribute to altered desmosome composition and turnover. Indeed, in early ultrastructural studies, it was suggested that the initial changes affect KIFs (20, 28). We here demonstrated that changes of the KIF cytoskeleton are visible in HaCaT keratinocytes starting at 60 min after application of autoantibodies. Reduced cell–cell adhesion is detectable even earlier, at least under mechanical stress, whereas endocytosis of Dsg3 is not apparent before 90 min of autoantibody exposure. Interestingly, these changes do not affect the amounts of long filaments running perpendicular to the cell membrane presumably anchoring desmosomes. Rather, the amounts of KIFs in basal and peripheral areas of the cell were reduced. These areas are known to be regions of KIF assembly. It is conceivable that binding of autoantibodies interferes with KIF cycling by limiting the assembly which, in the longer run, would then affect the entire network integrity. This is supported by the notion that the levels of CK14 are getting reduced after several hours. In this context, the thickening of remaining bundles after several hours may be interpreted as a cellular response to the failing adhesion (3). It has to be noted that in primary keratinocytes Dsg3 internalization was shown to occur after 60 min (29). This difference might be related to different maturation states of desmosomes in HaCaT vs. primary keratinocytes (30) or differences in the pathogenicity of the autoantibody fractions applied. For the current study, we relied on the HaCaT cell line for better expression efficiencies of two large proteins compared to primary keratinocytes.

Importantly, it cannot be ruled out that the overexpression approach applied in the current study affects the turnover of the respective molecules. For instance, it was shown that Dsg3 overexpression slows down its internalization and degradation and reduces monolayer fragmentation in response to pemphigus antibodies (24). In our study, the time frame of Dsg3 internalization of transfected and untransfected cells appeared to be at least largely similar as judged from the comparison of fixed, untransfected cells in Figure 1A and live cell imaging with Dsg3 overexpression in Figure 3A. Nevertheless, it is possible that Dsg3 internalization occurs faster in untransfected cells. Dsg3 internalization showed a similar time frame in HaCaT cells expressing CK5-YFP compared to cells with endogenous keratins only (compare Figure 3A; Figure S1A in Supplementary Material). This indicates that keratin overexpression does not alter Dsg3 internalization in response to PV-IgG but does not rule out an effect on keratin changes which may also occur faster in untransfected cells. At least under conditions in which both Dsg3 and keratins are overexpressed, the initial keratin changes precede the internalization of Dsg3.

It is unclear whether the KIFs in the basal cell periphery, which are depleted early in response to PV-IgG, are connected to desmosomes. Instead, the consequences on cell adhesion might be indirect. The desmosomal plaque proteins Dp and Pkp isoforms assemble in a juxtamembranous region together with already attached keratin filaments and are then transported to nascent desmosomes (31, 32). It is possible that this process is compromised by autoantibody-mediated reduced KIF assembly. Alternatively, the phenomenon of interdesmosomal widening may be connected to KIF alterations. It is known that the membranes of adjacent keratinocytes separate between the desmosomes at very early stages following autoantibody incubation (33, 34). As the KIF network is a major determinant of keratinocyte stiffness (35, 36), it is possible that a reduction in the cell periphery favors membrane separation in the interdesmosomal areas. Also, a weakening of the subplasmalemmal network of KIFs that may stabilize interdesmosomal membrane areas may contribute (37). This is supported by observations that p38MAPK inhibition, which blocks keratin retraction as detected in static images, is also reducing interdesmosomal widening (38). This suggests that the interdesmosomal membrane areas contribute to overall cell adhesion.

The observation that the alterations of the KIF cytoskeleton, in comparison to Dsg3 internalization, better correlate with loss of cell cohesion indicates a major contribution to PV-IgG-induced cell dissociation. This mechanism may contribute to the initial loss of cell cohesion that may be conferred through steric hindrance of Dsg3 interactions by PV-IgG. Indeed, it can be concluded from knockout studies that keratins are essential for strong cell–cell adhesion *in vivo* and *in vitro* (16, 39). Furthermore, it was shown that in cells lacking keratins, the forces by which two Dsg3 molecules trans-interact were reduced in a p38MAPK-dependent manner (40).

Based on these results, KIF changes appear not to be secondary to alterations of desmosomal adhesion molecules but to contribute to loss of cell cohesion even in the very early responses to PV-IgG challenge.

### Dsg3 Depletion and Keratin Changes May Be Regulated Independently

Given the temporal relationship of KIF alterations and Dsg3 internalization, it may be suggested that these two events are causally connected and Dsg3 membrane depletion is a consequence of altered KIF distribution. However, our results with the CK-1 inhibitor D4476 do not support this hypothesis. CK-1 inhibition resulted in a KIF phenotype resembling the effect of PV-IgG incubation. Under these conditions, no Dsg3 internalization or depletion was detectable whereas additional incubation with PV-IgG induced Dsg3 endocytosis. This supports a model in which Dsg3 membrane localization is independent from KIF changes. In line with these observations, keratinocytes derived from keratin knockout animals exhibit smaller desmosomes and reduced levels of some desmosomal molecules such as Dsg2 but have elevated levels of Dsg3 and unaltered plakoglobin content (16, 39, 40). Hence, the impact of keratins on desmosomal proteins is not uniformly affecting all molecules, which may be related to the observations that desmosomal molecules differentially contribute to cell cohesion (41, 42).

However, although the levels of Dsg3 in the membrane were unaltered, Dsg3 clustering was increased under conditions of D4476-mediated KIF reorganization. Together with the notion from previous data showing that Dsg3 mobility in the membrane is elevated in keratinocytes devoid of KIFs (40), this may indicate that Dsg3 is excluded from desmosomes and forms clusters that are subject to internalization. Indeed, such a model is supported by pulse-chase studies and live cell experiments (24, 43) of Dsg3 trafficking in response to PV-IgG. In this scenario, PV-IgG-induced Dsg3 endocytosis would require a first clustering step, which is normally suppressed by keratins or keratin insertion, and a second internalization step which is independent from keratins. For the latter step, lipid rafts may be essential (7).

Based on these observations, we inhibited Dsg3 endocytosis by interference with lipid raft composition through application of β-MCD. Interestingly, this abrogated Dsg3 endocytosis but did not prevent KIF alterations, indicating that these occur independently of Dsg3 internalization.

### Toward a Sequence of Structural Changes Resulting in Cell Dissociation

**Figure 7:**
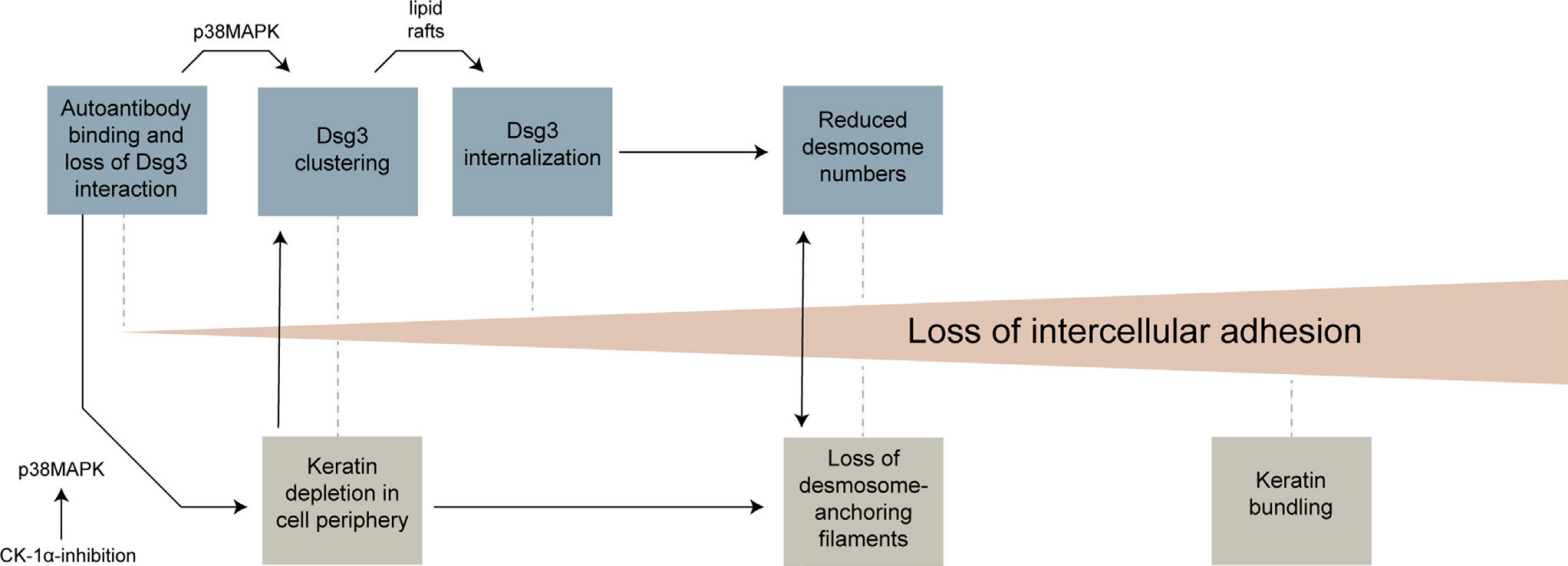
Overview of morphological changes leading to loss of keratinocyte adhesion in response to pemphigus vulgaris-IgG.

The results of this study and previous work from several groups can be integrated into a model in which distinct morphological hallmarks are merging to gradually increase cell dissociation (Figure 7). Anti-Dsg3 antibodies were shown to sterically inhibit Dsg3 trans-interactions (26, 44) which may contribute to initial loss of cell cohesion and represent a trigger for activation of p38MAPK (11). This, together with crosslinking effects of polyvalent anti-Dsg3 autoantibodies, induces an early clustering of Dsg3 molecules in the cell membrane (24, 45). Early alterations in keratin assembly may contribute to this effect, which also appear to be p38MAPK mediated and which were reproduced by CK-1 inhibition in this study. Dsg3 clustering is then followed by Dsg3 internalization, a process which is dependent on lipid rafts (7). Dsg3 endocytosis together with the internalization of other desmosomal molecules leads to destabilization of desmosomes. Importantly, internalization of desmosomal molecules affects both the assembly and the disassembly of desmosomes, leading to a reduction of desmosome size and numbers (4, 5). KIF loss or uncoupling from desmosomes, possibly bolstered by the altered keratin turnover, contribute to desmosome destabilization and *vice versa* (39, 40). Finally, the thickening of keratin filaments observed at later stages may represent a general cellular stress response or can be interpreted as futile attempt to strengthen cell–cell adhesion by better anchoring the remaining desmosomes. Other mechanisms so far described in pemphigus such as diverse alterations in intracellular signaling, the occurrence of non-desmosomal autoantibodies, or the genetic background (6), may contribute by modulating these morphological changes. It is conceivable that several of these steps need to occur to induce full loss of cell cohesion and blistering. Depending on the experimental setup (e.g., IgG fraction, Ca^2+^ dependency of desmosomes, model system), some of these steps may be more important than others. This may explain some of the controversies existing in the field of pemphigus research.

## Ethics Statement

The sera used in the present study were collected in accordance with the recommendations of ethic committee of University of Lübeck, AZ12-178; name of project: Autoantikörperreaktivität und Pathophysiologie bei blasenbildenden Autoimmunerkrankungen (Pemphigoid und Pemphigus).

## Author Contributions

ES performed experiments and analyzed data. MR created constructs. FV and CS supervised experiments and analyzed data. JW and VS analyzed data and interpreted results. VS designed the study. ES and VS wrote the manuscript.

## Conflict of Interest Statement

The authors declare that the research was conducted in the absence of any commercial or financial relationships that could be construed as a potential conflict of interest.

## Supplementary Material

Video S1 HaCaT-CK5 cells transfected with pDest-mDsg3-mCherry and incubated with PV1-IgG for 120 min. Images were acquired every 2 min.

Video S2 60 min D4476 incubation in HaCaT-CK5 cells transfected with pDest-mDsg3-mCherry followed by 180 min of PV2-IgG incubation. Images were collected every 2 min.

Figure S1 **(A)** HaCaT wild-type keratinocytes showed a similar time course of Dsg3 internalization as HaCaTs expressing cytokeratin5 (CK5)-yellow fluorescent protein (*n* = 4, **p* < 0.05). **(B)** HaCaT-CK5 lysates were processed into a TX-100 insoluble and soluble pool showing p38MAPK activation (*n* = 4). **(C,D)** p38MAPK activation and protein levels in HaCaT-CK5 cells after the indicated treatment which correlate to Figures 4D,E. Cells were processed for Western blotting as SDS lysates [panel **(C)**: *n* = 4, panel **(D)**: *n* = 3–5].
