# Supplementary figures and images for: Keratin Retraction and Desmoglein3 Internalization Independently Contribute to Autoantibody-Induced Cell Dissociation in Pemphigus Vulgaris
Source: Front Immunol. 2018 Apr 25;9:858. doi: 10.3389/fimmu.2018.00858 (PMC5996934; doi:10.3389/fimmu.2018.00858)

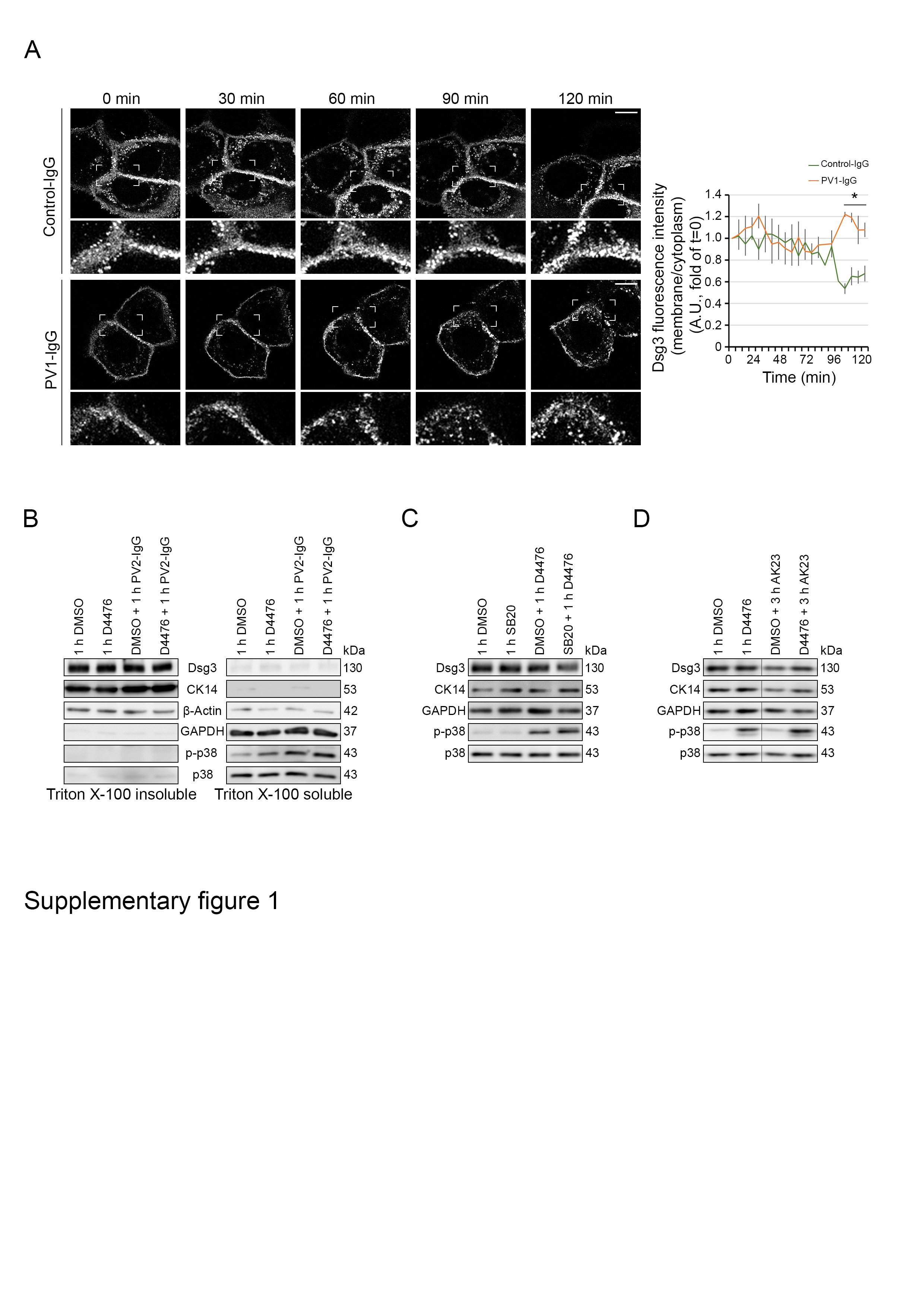

Supplement: Figure S1 — (A) HaCaT wild-type keratinocytes showed a similar time course of Dsg3 internalization as HaCaTs expressing cytokeratin5 (CK5)-yellow fluorescent protein (n = 4, *p < 0.05). (B) HaCaT-CK5 lysates were processed into a TX-100 insoluble and soluble pool showing p38MAPK activation (n = 4). (C,D) p38MAPK activation and protein levels in HaCaT-CK5 cells after the indicated treatment which correlate to Figures 4D,E. Cells were processed for Western blotting as SDS lysates [panel (C): n = 4, panel (D): n = 3–5]. [file image_1.tif]
